# Adjuvant 904 nm Gallium Arsenide Laser Therapy Enhances Healing and Reduces Bacterial Burden in Chronic Wounds: A Randomized Controlled Trial

**DOI:** 10.1111/wrr.70224

**Published:** 2026-10-22

**Authors:** Valéria da Silva Baracho, Natália Cristina da Silva, Lourdes Fernanda Godinho, Cláudia Aparecida Fernandes Cordeiro, Lorranne Ferreira Custodio, Brendha Lorrany Alves, Luiz Otávio Lopes Teixeira, Rodrigo Pizzini de Souza, Marco Antônio Schetino, Vinícius de Oliveira Ottone, Murilo Xavier de Oliveira, Thabata Coaglio Lucas, Kinulpe Honorato Sampaio

**Affiliations:** ^1^ Programa de Pós‐Graduação Em Ciências da Saúde Universidade Federal dos Vales do Jequitinhonha e Mucuri Diamantina Minas Gerais Brazil; ^2^ Gerência de Vigilância Epidemiológica da Prefeitura Municial de Belo Horizonte Belo Horizonte Minas Gerais Brazil; ^3^ Faculdade de Ciências Biológicas e da Saúde Universidade Federal dos Vales do Jequitinhonha e Mucuri Diamantina Minas Gerais Brazil; ^4^ Santa Casa de Caridade de Diamantina Diamantina Minas Gerais Brazil; ^5^ Faculdade de Medicina Universidade Federal dos Vales do Jequitinhonha e Mucuri Diamantina Minas Gerais Brazil

**Keywords:** diabetic ulcers, photobiomodulation, venous ulcers, wound healing

## Abstract

Chronic wounds, characterized by prolonged healing times and high complication rates, present significant challenges to healthcare systems. This randomized, double‐blind, placebo‐controlled clinical trial evaluated the adjunctive effect of 904 nm gallium arsenide (GaAs) laser therapy in the treatment of chronic venous and diabetic ulcers. The study adhered to the CONSORT 2025 guidelines to ensure transparency and quality in reporting. Seventy patients were randomly assigned to two groups: active laser (AL) group (laser + standard care) and placebo laser group (PL) (simulated laser + standard care). All participants were followed twice a week until complete wound healing or a maximum of 15 sessions. Outcomes assessed included wound area reduction, microbial load and histological analysis. Results showed that the AL group had a 53% reduction in wound area, while the PL group had a 9% reduction (*p* < 0.001), with complete closure observed in two AL patients. Laser therapy also significantly reduced bacterial colonization, particularly, of *Pseudomonas aeruginosa* , *Escherichia coli* and *Proteus mirabilis* (*p* ≤ 0.007). Histological analysis demonstrated enhanced angiogenesis, reduced inflammatory infiltrate and improved collagen organization at the wound edges in the AL group (*p* ≤ 0.042). These findings demonstrate that 904 nm GaAs laser therapy accelerates healing, reduces microbial burden and promotes favourable tissue remodelling. The integrative approach, combining clinical, microbiological and histological assessments, underscores its potential as a safe and effective adjunct in chronic wound management, offering a promising strategy to improve outcomes and reduce healthcare burdens.

## 1 Introduction

Chronic wounds represent a significant public health concern and remain a challenge for healthcare systems due to the complexity of their treatment. These wounds are characterized by a prolonged and difficult healing process, with restoration of the skin's anatomical and functional integrity taking longer than 3 months [1, 2]. In addition to delayed healing, chronic wounds are associated with high recurrence rates and frequently lead to complications such as infection, necrosis and limb amputation [3, 4].

Wound healing is a physiological process involving biochemical, cellular and molecular mechanisms and relies on the coordinated interaction between cells and inflammatory mediators to replace or restore damaged tissue. While acute wounds follow the normal stages of healing (haemostasis, inflammation, proliferation and remodelling), chronic wounds fail to progress through these phases and remain stalled in the inflammatory stage [5]. This stagnation compromises the efficacy of conventional wound care. The chronic nature of such lesions is often associated with intrinsic pathological or physiological dysfunctions, including age, smoking, impaired mobility, sensory alterations, neuropathy, poor nutritional status, chronic diseases, vascular impairment, infections or neoplastic events [6].

Among the most common chronic wounds are diabetic and venous ulcers, particularly, prevalent among older adults [7]. According to the literature, complications related to diabetes affect between 40 and 60 million people worldwide and approximately 20% of moderate to severe ulcer cases of diabetic foot require amputation, severely impacting the quality of life of affected individuals [8]. Regarding vasculogenic ulcers, venous insufficiency accounts for approximately 80% of cases, while 10%–20% are arterial in origin and 10%–15% are of mixed aetiology [9]. These wounds place a substantial financial burden on healthcare systems, reduce individuals' working capacity and contribute to increased hospitalizations due to complications such as infection [10].

Another critical factor in the persistence of chronic wounds is infection, particularly, the formation of biofilms, which interfere with healing and prolong the inflammatory phase [11]. Although all wounds are susceptible to microbial colonization, individuals with chronic wounds often have a compromised immune system, which hinders the elimination of microbial load. This immunological burden facilitates the transition from colonization to critical colonization and ultimately to infection [12]. Moreover, poor hygiene and inadequate wound care promote microbial proliferation. Early diagnosis of infections is, therefore, crucial for preventing complications, minimizing their consequences and optimizing wound healing.

Traditional treatments for chronic wounds, including wound cleansing, debridement of necrotic tissue, application of advanced dressings, infection control and management of underlying conditions such as diabetes and venous insufficiency, remain the cornerstone of clinical practice [13]. However, the conventional therapies often yield suboptimal outcomes due to the inherently protracted and inefficient healing process, highlighting the urgent need for novel therapeutic strategies [14]. So, photobiomodulation (PBM) has gained increasing attention as a promising adjunctive treatment [15, 16, 17]. It has shown potential in reducing microbial load [18], offering analgesic and anti‐inflammatory effects [19, 20, 21]. Thus, PBM has emerged as an innovative, non‐pharmacological, non‐invasive and cost‐effective therapeutic approach for wound management, particularly, through the use of LED and laser devices [18].

Laser devices emit coherent low‐intensity light in red or near‐infrared wavelengths. Due to their coherent and intense nature, lasers interact specifically with biological tissues and act more effectively on viable tissue, with limited direct effect on coagulative necrosis, characterized as cell death caused primarily by impaired blood supply and cell function in pathological conditions [19]. When combined with conventional dressings, laser therapy represents a promising strategy, with studies reporting its beneficial effects in clinical wound management. This includes modulation of the inflammatory response, stimulation of cellular metabolism, improved blood flow and accelerated healing [20, 21].

The gallium arsenide (AsGa) laser, a semiconductor laser that emits infrared light typically at 904 nm, has demonstrated experimental potential for promoting epithelial cell regeneration [22, 23], as well as for stimulating various other cell types [24, 25]. Despite the promising evidence, most studies involving the AsGa laser remain at the experimental or case‐report level. To date, only one recent clinical trial has evaluated the effects of AsGa laser therapy (904 nm) on the healing of diabetic foot ulcers [26].

In this context, the present study aimed to evaluate the adjunctive effect of 904 nm AsGa laser therapy in the treatment of chronic wounds of venous and diabetic origin through a randomized, double‐blind, placebo‐controlled clinical trial. Notably, this is the first clinical trial to assess the effects of this therapy on clinical wound characteristics, microbial reduction and direct histological analysis of the healing process.

## 2 Materials and Methods

### 2.1 Study Design

This study was designed as a randomized, double‐blind, placebo‐controlled clinical trial, conducted at a multidisciplinary outpatient facility located in the Jequitinhonha Valley, Minas Gerais, Brazil. Participants were randomly allocated into two groups: (1) the active laser (AL) group, which received PBM therapy with laser in addition to standard wound care and (2) the placebo laser (PL) group, which underwent a simulated laser intervention alongside standard care. The primary outcome was the percentage reduction in wound area over time. Secondary outcomes included clinical wound improvement (assessed by the presence of granulation tissue and absence of signs of infection), histopathological changes in scar tissue (evaluated using a standardized histological scoring system) and bacterial reduction, quantified by polymerase chain reaction (PCR) analysis.

Ethical approval was granted by the Ethics and Research Committee of the Federal University of the Jequitinhonha and Mucuri Valleys (UFVJM) (protocol number 5.331.786). All participants provided written informed consent prior to enrollment. Data collection took place from June 2022 to February 2024. This clinical trial followed the recommendations of the CONSORT 2025 statement [27] and was registered in the Brazilian Clinical Trials Registry (REBEC) under the number RBR‐8xd262t on 10 May 2022. The CONSORT checklist and flow diagram were used to ensure the complete and transparent presentation of the study design, methodology, results and interpretation.

### 2.2 Sample Size Calculation

Sample size calculation was performed using G*Power software (version 3.1.9.7, Heinrich Heine University, Düsseldorf, Germany). A two‐tailed *t*‐test for independent means was used, assuming a medium to large effect size (Cohen's *d* = 0.68), an alpha level of 0.05 and a power of 80%. The estimated minimum sample size was 68 participants (34 per group). To account for potential dropouts and protocol deviations (~15%), the sample size was increased to 78 participants, equally randomized into two groups (*n* = 39). Due to differential attrition, the final sample comprised 32 participants in the placebo group and 38 in the intervention group, maintaining the minimum required sample size and statistical power.

### 2.3 Procedure

The study population consisted of patients with chronic wounds and a confirmed diagnosis of systemic arterial hypertension and/or diabetes mellitus, selected through medical and nursing screening. To ensure eligibility, only patients with a confirmed clinical diagnosis of chronic lower limb ulcers lasting more than 6 weeks, of venous and neuropathic aetiology, as documented in medical records and confirmed by evaluation performed by trained medical and nursing staff, were included. The ulcer aetiology was determined based on physical examination, clinical history and, when necessary, complementary tests such as Doppler ultrasound. Inclusion criteria comprised individuals of any sex, aged 18 years or older, presenting with chronic lower limb wounds and agreeing to undergo swab and biopsy collection at two different time points during the intervention.

Exclusion criteria included: other types of skin lesions; pregnancy; current use of immunosuppressants or chronic corticosteroid therapy; patients undergoing cancer treatment; wounds exhibiting active infection or presenting with over 25% necrotic tissue coverage; wounds in the final epithelialization stage; and individuals with photosensitivity or any known adverse reaction to light exposure. These criteria were applied to minimize the risk of adverse events and ensure a higher probability of treatment effectiveness.

A total of 78 participants were randomized: 39 were assigned to the AL group and 39 to the PL group. The placebo procedure used a device identical in appearance, sound and operation to the AL but without therapeutic energy emission. To ensure blinding, both participants and researchers were unaware of group allocation throughout the study. Blinding integrity was periodically assessed by asking participants and staff about their perceptions of group assignment, confirming that neither group could reliably identify the treatment received. This rigorous double‐blind design ensured an unbiased assessment of treatment outcomes.

Three participants in the PL group were lost to follow‐up (one due to transportation issues and two due to treatment dropout). Additionally, five participants were excluded due to non‐adherence to wound care protocols. Consequently, data from 70 participants were analyzed, 32 in the AL group and 38 in the PL group.

Participants were randomized in a 1:1 ratio using a computer‐generated sequence (random.org), created in advance by an independent researcher not involved in recruitment or intervention. To ensure allocation concealment, each participant received a unique code during the initial assessment, which was presented to the responsible researcher. This researcher then consulted a pre‐generated list, strictly following the sequential order and maintaining blinding. Randomization was implemented using opaque, sealed and sequentially numbered envelopes prepared by a third party. The trial was conducted in a double‐blind manner: both participants and evaluators were unaware of group allocations. Follow‐up sessions occurred twice a week until complete healing or the limit of 15 sessions was reached.

The laser device used was a AsGa therapeutic laser, model Mid Laser (Sweden), featuring 12 simultaneous emitting diodes, with a wavelength of 904 ± 10 nm, frequency of 200 Hz and output power of 60 mW [28]. The dosage of 8 J/cm^2^ was adopted based on prior research that demonstrated its efficacy for diabetic wound healing [26]. To prevent cross‐contamination, the laser was sanitized with 70% alcohol and covered with PVC film before each session. Protective goggles were worn by both the patient and the researcher during all applications.

To standardize care across both groups, wound assessments and dressing selection were performed by a qualified professional. Variability in the choice of therapy was controlled through standardized clinical criteria and based on the characteristics of each wound, ensuring consistency in treatment across groups. The protocol included cleansing wounds with warm 0.9% saline solution prior to treatment, followed by the application of appropriate conventional dressings. These dressings served various purposes: hydration (essential fatty acids), slough debridement (hydrogel with alginate), maintenance of a moist wound environment and stimulation of healing (plain hydrogel), exudate control (alginate foam), biofilm management (activated charcoal) and grafting (alginate sheet). In cases of venous ulcers, compression therapy using an Unna boot was also employed to reduce oedema.

### 2.4 Data Collection and Sampling

A questionnaire was administered to collect sociodemographic and clinical data. Sociodemographic variables included sex, age and race. Clinical data comprised comorbidities and wound characteristics such as aetiology, duration, odour, maceration, oedema, granulation tissue, slough, epithelialization, pruritus and pain. The anatomical location of each ulcer was prospectively recorded during the clinical evaluation. Although ulcer location was documented for all participants, it was not considered a stratification variable in the present trial.

Wound area was monitored throughout the study as a key indicator of healing. A 0–15 cm Vernier calliper was used, carefully positioned above the wound without direct skin contact, following the wound's contours as precisely as possible. Considering that venous ulcers often present irregular edges and are located on non‐flat surfaces, the measurement was supplemented with standardized photographic documentation. Images were captured using a Canon EOS Rebel XS camera (10.1 megapixels, 18–55 mm lens) and wound area was quantified using ImageJ software (US National Institutes of Health, Bethesda, MD), allowing for greater accuracy and reproducibility, especially in wounds with complex topography.

Swab samples were aseptically collected from both groups using sterile rayon swabs with plastic shafts, which were premoistened with sterile 0.9% saline solution to assess microbial presence using real‐time polymerase chain reaction (RT‐PCR). Collection was performed after wound cleansing with saline, applying the Levine technique [29], targeting viable granulation tissue at the wound centre. The swab samples were stored at −80°C until molecular analysis.

Following, tissue biopsies were performed under sterile conditions with local anaesthesia (2% lidocaine), administered by a qualified surgeon. Specimens were taken from both the wound edge and centre using a 4 mm punch biopsy tool, then fixed in 4% paraformaldehyde for 24 h and subsequently transferred to 70% ethanol to evaluate tissue healing through histological analysis.

### 2.5 RT‐PCR

Microbiological analysis was conducted via RT‐PCR. Total RNA was extracted from swab samples using TRI Reagent (Sigma). Reverse transcription was performed in a thermal cycler (StepOne PCR System, Applied Biosystems, Foster City, CA, USA) with the following conditions: 10 min at 42°C, 40 min at 55°C and 10 min at 70°C. The reaction mix contained 9 μL of sample, 4 μL Script RT buffer, 4 μL RNase‐free water, 1 μL dNTP mix, 1 μL DTT, 0.5 μL random primer, 0.5 μL RNase inhibitor and 0.5 μL Script Reverse Transcriptase (Cellco).

RT‐PCR was performed using primers targeting: *Pseudomonas aeruginosa* , sense 5′ → 3′ AGG TCG GAG CTG TCG TAC TC and antisense 5′ → 3′ ACC CGA ACG CAG GCT ATG; *Escherichia coli* , sense 5′ → 3′ TGG GAG CGA AAA TCC TG and antisense 5′ → 3′ CAG TAC AGG TAG ACT TCT G; *Klebsiella oxytoca* , sense 5′ → 3′ GAT ACG GAG TAT GCC TTT ACG GTG and antisense 5′ → 3′ TAG CCT TTA TCA AGC GGA TAC TGG and *Proteus mirabilis* , sense 5′ → 3′ CCA TCA GAT TAT GTC ATT CAA and antisense 5′ → 3′ GAG GAA AAT GCA ATT TAT CTT TA. The reaction mix included 5 μL SYBR Green PCR Master Mix (Sigma), 0.4 μL of each primer (10 μM), 4 μL DPEC water and 1 μL cDNA. The amplification protocol consisted of an initial denaturation at 95°C for 3 s and annealing at 60°C for 20 s. A melt curve analysis was conducted to verify amplification specificity. Negative controls were prepared by replacing cDNA with RNase‐free water and no amplification was observed in control reactions without reverse transcriptase. All reactions were run in duplicate.

### 2.6 Histological Analysis

The tissue specimens were fixed in formalin, embedded in paraffin and sectioned at 5 μm thickness. For each biopsy, two paraffin blocks were prepared, corresponding to the central region and the wound edge. Two to three histological sections from each patient were analyzed. Sections were stained with haematoxylin and eosin (H&E) and Masson's trichrome (MT). All histological slides were stained simultaneously under identical experimental conditions to minimize staining variability among samples. To assess the tissue‐level healing response, biopsies from seven patients in each treatment group were selected. To minimize bias, participants were matched based on similar baseline characteristics, including initial wound size, age and lesion aetiology.

Histopathological evaluation was performed directly on the complete histological sections under light microscopy by two independent blinded examiners using predefined semi‐quantitative criteria adapted from the scoring system originally proposed by Abramov et al. [30] and subsequently reviewed by Gupta and Kumar [31]. Because no universally accepted histological scoring system for wound healing currently exists, this modified semi‐quantitative approach was adopted to comprehensively evaluate the principal histopathological events involved in tissue repair. Both examiners were blinded to treatment allocation and biopsy time point (baseline or post‐treatment). Whenever disagreement occurred, the slides were independently reviewed by a third blinded examiner until consensus was reached.

Histological scoring was performed by systematic examination of the entire histological section. Low magnification (4× objective) was initially used to evaluate the overall architecture of the tissue and obtain a global assessment of the healing process. Higher magnification (40× objective) was subsequently used to confirm the identity of specific cellular or tissue structures, including inflammatory infiltrates, fibroblasts and newly formed blood vessels. The final histological score represented the overall histopathological impression of the entire tissue section rather than isolated microscopic fields.

The semi‐quantitative scoring system ranging from 0 to 3 for four key parameters of wound repair: inflammatory infiltrate, angiogenesis, fibroblast cellularity and collagen organization (Table 1). For all variables, a score of 3 represented the least favourable histological condition (absence of tissue repair or persistence of pathological features), whereas a score of 0 represented the most advanced stage of tissue repair (complete or near‐complete restoration of tissue architecture). Intermediate scores (1 and 2) represented progressive stages of healing according to the degree of tissue organization and regeneration.

**TABLE 1:** Evaluation scores used for H&E and TM staining, Diamantina, MG, Brazil, 2025.

Histological parameter	Score 3	Score 2	Score 1	Score 0
Inflammatory infiltrate	Abundant inflammatory infiltrate	Moderate infiltrate	Mild infiltrate	Absence of inflammatory infiltrate
Angiogenesis	No newly formed vessels	Sparse neovascularization	Moderate neovascularization	Abundant, well‐organized vascularization
Fibroblast cellularity	Sparse immature fibroblasts	Moderate cellularity	High proliferative fibroblast population	Mature, organized fibroblasts
Collagen organization	Absent collagen	Sparse, disorganized collagen	Moderate deposition with partial organization	Dense, well‐organized collagen bundles with intense staining

Abbreviations: H&E: Haematoxylin and eosin; TM: Masson's trichrome.

Inflammatory infiltrate was graded according to the density of acute and chronic inflammatory cells throughout the wound bed, ranging from abundant inflammatory infiltrate (Score 3) to complete resolution of inflammation (Score 0). Angiogenesis was evaluated based on the abundance, distribution and maturation of newly formed blood vessels within the granulation tissue. Scores ranged from the absence of neovascularization (Score 3), through sparse (Score 2) and moderate (Score 1) vascular proliferation, to abundant and well‐organized vascularization consistent with advanced tissue repair (Score 0). Fibroblast cellularity was assessed considering both fibroblast density and morphological maturation. Higher scores represented sparse cellularity with predominantly immature, disorganized fibroblasts, whereas lower scores indicated progressive fibroblast maturation and organization, culminating in mature spindle‐shaped fibroblasts arranged in an organized extracellular matrix (Score 0). Collagen deposition was evaluated using Masson's trichrome staining considering three complementary histopathological features: (i) the amount of collagen deposition, (ii) the architectural organization of collagen bundles and (iii) the staining characteristics of the extracellular matrix. Higher scores represented absent or sparse collagen deposition with poorly organized extracellular matrix, whereas lower scores indicated progressively denser collagen deposition, greater bundle organization and stronger staining intensity, consistent with advanced extracellular matrix remodelling. Masson's trichrome staining is widely employed for the histopathological assessment of connective tissue remodelling because it differentiates collagen fibres from surrounding tissues and provides qualitative information regarding extracellular matrix organization. During wound healing, collagen fibres typically evolve from sparse, loosely organized, lightly stained fibres deposited during the proliferative phase towards denser, more organized bundles exhibiting greater staining intensity during tissue remodelling. Accordingly, the collagen score considered not only collagen deposition but also the organization and staining characteristics of the extracellular matrix.

Representative images were subsequently acquired during a single imaging session using a BX53F microscope (Olympus, Japan) under identical acquisition parameters, including illumination intensity, exposure time, white balance, camera settings and objective magnification, thereby minimizing technical variability between groups. Representative photomicrographs were acquired only after completion of the histopathological evaluation and are presented exclusively for illustrative purposes. They were not used for histological scoring or quantitative analyses.

### 2.7 Statistical Analysis

Categorical variables were presented as absolute and relative frequencies and compared using the *χ* ^2^ test or Fisher's exact test, when any parameter in one of the groups had *n* < 5. Continuous variables were expressed as mean ± standard deviation. The Shapiro–Wilk test was applied to assess normality, followed by the non‐parametric Mann–Whitney *U* test for comparisons between groups. Paired analyses of microbiological and clinical wound characteristics before and after treatment in both the PL and AL groups were performed using McNemar's test or Paired *t*‐test. All statistical analyses were conducted using Jamovi software (version 2.3), with statistical significance set at *p* < 0.05.

## 3 Results

### 3.1 Patient Characteristics

Table 2 presents the distribution of variables related to patient characteristics. The mean age of participants was 66.1 ± 16.7 years in the AL group and 65.4 ± 14.4 years in the PL group, with 32 individuals (45.7%) aged over 70 years. Forty participants (57.1%) were male and 39 (55.7%) self‐identified as mixed race (parda). No significant differences were found between groups regarding sex (*p* = 0.406) or age (*p* = 0.645). The majority of patients (56, 80%) presented with venous ulcers, while 14 (20%) had neuropathic ulcers. Hypertension was the most prevalent comorbidity, affecting 48 participants (68.6%), followed by Type 2 diabetes mellitus (DM2), reported in 32 cases (45.7%). There were no statistically significant differences between groups concerning the prevalence of comorbidities. Likewise, no significant difference was observed in baseline wound size, which averaged 38.3 ± 16.2 cm^2^ in the AL group and 48.4 ± 16.1 cm^2^ in the PL group (*p* = 0.070). Ulcers were predominantly located in the distal lower limb, including the anterior leg, medial and lateral malleolar regions, dorsum and plantar surface of the foot, ankle and toes, consistent with the clinical characteristics of chronic venous and diabetic neuropathic ulcers.

**TABLE 2:** Characterization of the sample regarding sociodemographic and clinical aspects. Diamantina, MG, Brazil, 2025.

Variables	Al (*n* = 38)	Pl (*n* = 32)	*p*	Test
Sex				
Female	18 (47.4%)	12 (37.5%)	0.406	*χ* ^2^
Male	20 (52.6%)	20 (62.5%)		
Age	66.1 ± 2.7	65.4 ± 2.6	0.645	*U*
Race				
Mixed‐race	21 (55.3%)	18 (56.3%)	0.765	*χ* ^2^
Black	15 (39.5%)	11 (34.4%)		
White	2 (5.3%)	3 (9.4%)		
Wound origin				
Venous	30 (78.9%)	26 (81.3%)	0.810	*χ* ^2^
Neuropathic	8 (21.1%)	6 (18.8%)		
Comorbidities				
Hypertension	24 (63.2%)	24 (75.0%)	0.288	*χ* ^2^
Type 2 diabetes	21 (55.3%)	11 (34.4%)	0.081	*χ* ^2^
Lesion size (cm^2^)	38.3 ± 16.2	48.4 ± 16.1	0.070	*U*

*Note:* Data are presented as absolute numbers (%) or mean ± standard error.

Abbreviations: Al: active laser; Pl: placebo laser; *U*: Mann–Whitney test; *χ* ^2^: chi‐square test.

*p*‐Values < 0.05 were considered statistically significant.

### 3.2 AsGa Laser Accelerated Wound Healing and Improved Clinical Parameters

Throughout the treatment period, a significant reduction in wound area was observed following AsGa laser therapy. Wounds in the PL group showed an average reduction of approximately 9%, whereas those in the AL group exhibited a markedly greater reduction of 53%, with final wound areas reaching 91.2% ± 7.2% and 47.4% ± 5.5%, respectively.

Table 3 presents the comparison between the control and treatment groups at baseline and after 15 treatment sessions. Both groups showed significant clinical improvement in wound characteristics. In the PL group, significant improvement was observed in four parameters: foetid odour (*p* = 0.002), macerated skin (*p* = 0.020), oedema (*p* = 0.007) and slough (*p* = 0.002). In contrast, the AL group demonstrated statistically significant improvement across all eight evaluated variables, with *p*‐values < 0.001 for each parameter. Figure 1 illustrates the healing progression of three patients from the PL group and three from the AL group, highlighting more rapid wound resolution in patients treated with AsGa laser. Notably, complete wound closure was achieved in two patients from the AL group.

**TABLE 3:** Macroscopic wound parameters before and after treatment in the control and treated groups. Diamantina, Minas Gerais, Brazil, 2025.

Variables	Groups	Before, *n* (%)	After, *n* (%)	*p* ^a^
Foetid odour	Placebo laser	16 (50.0%)	26 (81.2%)	**0.002**
	Active laser	23 (60.5%)	34 (89.5%)	**< 0.001**
Macerated skin	Placebo laser	16 (50.0%)	23 (71.9%)	**0.020**
	Active laser	21 (55.3%)	34 (89.5%)	**< 0.001**
Oedema	Placebo laser	5 (15.6%)	14 (43.7)	**0.007**
	Active laser	14 (15.6%)	26 (68.4%)	0.670
Slough tissue	Placebo laser	5 (15.6%)	15 (46.9%)	**0.002**
	Active laser	4 (10.5%)	31 (84.2%)	**< 0.001**
Pruritus	Placebo laser	16 (50.0%)	21 (65.6%)	0.059
	Active laser	19 (50.0%)	32 (84.2%)	**< 0.001**
Granulation tissue	Placebo laser	16 (50.0%)	21 (65.6%)	0.059
	Active laser	14 (36.8%)	1 (2.6%)	**< 0.001**
Pain	Placebo laser	6 (18.7%)	9 (28.1%)	0.180
	Active laser	3 (7.9%)	28 (73.7%)	**< 0.001**
Epithelial tissue	Placebo laser	22 (68.7%)	11 (34.4%)	**0.002**
	Active laser	25 (65.8%)	5 (13.1%)	**< 0.001**

*p*‐values < 0.05 were considered statistically significant (in bold).

^a^ McNemar test, *n* = 32 in the placebo laser and *n* = 38 in the active laser.

**FIGURE 1:**
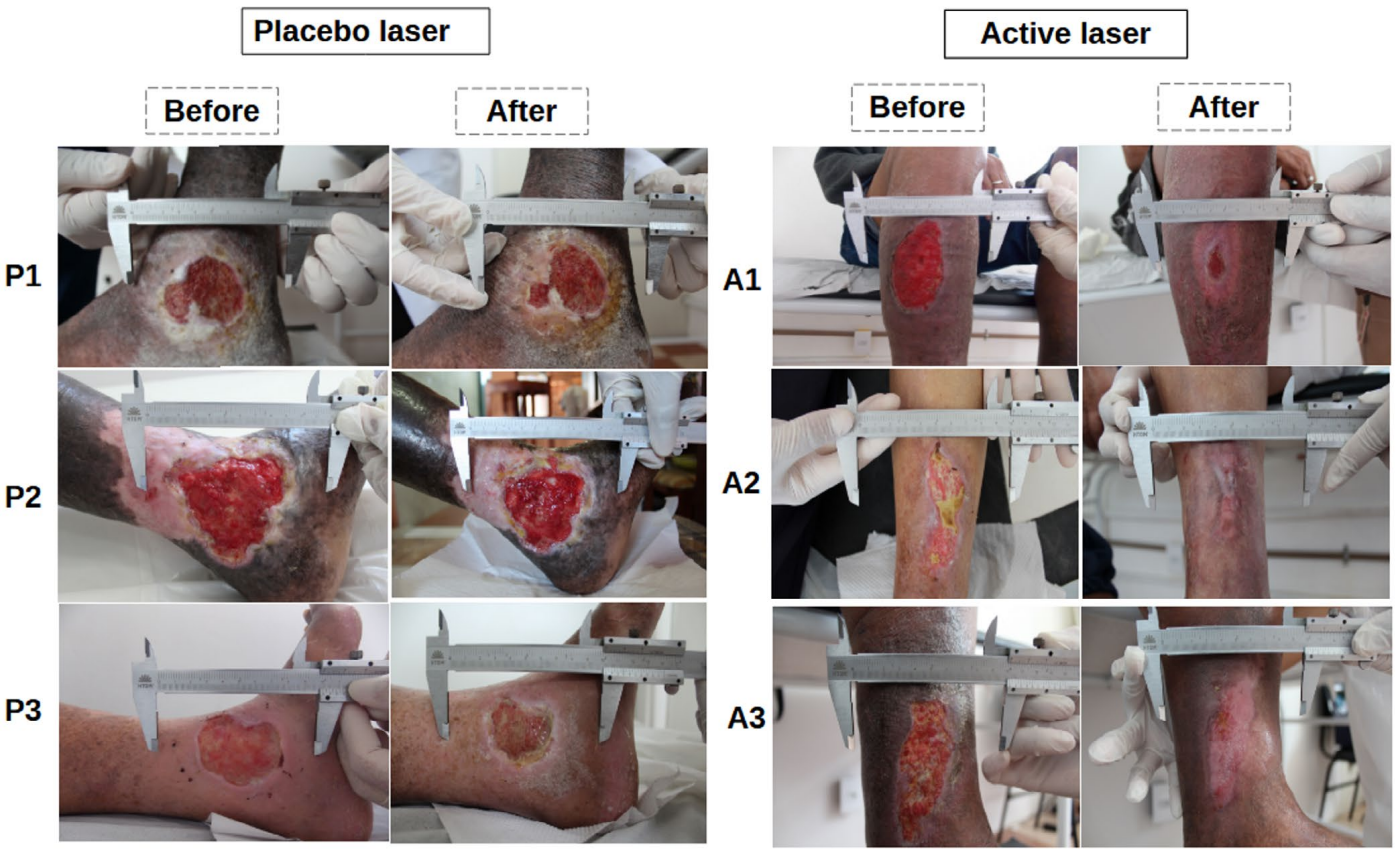
Chronic wound treatment with AsGa laser before and after 15 sessions. A: active laser group; P: placebo laser group.

### 3.3 AsGa Laser Reduced the Number of Infected Wounds

This study assessed the presence of four common Gram‐negative bacterial species typically found in infected wounds using RT‐PCR (Table 4). Sampling was performed via swabs aseptically collected after wound cleansing. A high prevalence of infected wounds was identified: 81.5% of wounds in the PL group tested positive for at least one of the bacteria analyzed (26 out of 32 patients), compared to 79% in the AL group (30 out of 38 patients). *P. aeruginosa* was the most prevalent pathogen in both groups. Laser therapy significantly reduced the presence of *P. aeruginosa* (*p* = 0.005), *E. coli* (*p* = 0.007) and *P. mirabilis* (*p* < 0.001) in the AL group. In the control group, a significant reduction was observed only for *P. mirabilis* . Although *K. oxytoca* showed a slight decrease in both groups, this change was not statistically significant (*p* = 0.317 and *p* = 1.000, respectively). Given that *K. oxytoca* was the least prevalent species, the limited sample size may have precluded the detection of significant differences.

**TABLE 4:** Number of positive cases before and after treatment with laser therapy. Diamantina, Minas Gerais, Brazil, 2025.

Microorganismos	Groups	Before, *n* (%)	After, *n* (%)	*p* ^a^
*Pseudomonas aeruginosa*	Placebo laser	20 (62.5%)	20 (62.5%)	1.000
	Active laser	20 (52.6%)	9 (23.7%)	**0.005**
*Escherichia coli*	Placebo laser	8 (25%)	10 (31.2)	0.763
	Active laser	15 (39.5)	6 (15.8)	**0.007**
*Klebsiella oxytoca*	Placebo laser	6 (18.7)	5 (15.6)	0.317
	Active laser	2 (5.3)	2 (5.3)	1.000
*Proteus mirabilis*	Placebo laser	7 (21.9)	2 (6.2)	**< 0.001**
	Active laser	5 (13.1)	3 (7.9)	**< 0.001**

*p*‐Value < 0.05 were considered statistically significant (in bold).

^a^ McNemar test, *n* = 32 in the placebo laser and *n* = 38 in the active laser.

### 3.4 AsGa Laser Stimulated Angiogenesis, Collagen Deposition and Reduced Inflammatory Infiltrate at Wound Edges

To assess the healing process at the cellular and tissue levels, biopsies from the wound centre and edge were obtained from seven patients per group, selected based on comparable wound characteristics. Initial wound areas were similar between groups (18.2 ± 4.6 cm^2^ in the PL group vs. 18.3 ± 3.5 cm^2^ in the AL group; *p* = 0.9932), as were mean patient ages (66.9 ± 5.5 years in PL vs. 63.9 ± 7.8 years in AL; *p* = 0.7590). Lesion aetiology also showed no significant differences, with venous ulcers predominating (85.7% in PL vs. 71.4% in AL) and a smaller proportion of neuropathic ulcers (14.3% vs. 28.6%, respectively; Fisher's exact test *p* > 0.9999).

Table 5 presents the histological scoring data, where lower scores indicate improvement in the assessed parameters. A significant reduction in vascular density score was observed at the wound edge in the AL group (*p* = 0.0127), suggesting enhanced angiogenesis induced by the laser (Figure 2A). A concurrent reduction in inflammatory infiltrate was also seen at the wound edge (*p* = 0.0103), indicating an anti‐inflammatory effect of laser therapy (Figure 2B). No significant differences were observed in the wound centre for either parameter.

**TABLE 5:** Evaluation of the semi‐quantitative histological scores of wound edge and centre, before and after treatment in the placebo laser and active laser groups. Diamantina, Minas Gerais, Brazil, 2025.

Variables	Location	Groups	Before (mean ± standard error)	After (mean ± standard error)	*p* ^a^
Angiogenesis	Border	Placebo laser	1.00 ± 0.71	1.00 ± 0.71	> 0.9999
		Active laser	1.50 ± 0.84	0.33 ± 0.52	**0.0127**
	Centre	Placebo laser	1.42 ± 0.79	1.14 ± 0.69	0.6036
		Active laser	0.60 ± 0.89	0.40 ± 0.55	0.7489
Inflammatory infiltrate	Border	Placebo laser	2.88 ± 0.45	2.88 ± 0.45	1.00
		Active laser	2.83 ± 0.41	1.50 ± 0.55	**0.0103**
	Centre	Placebo laser	2.57 ± 0.79	2.42 ± 0.79	0.7882
		Active laser	2.60 ± 0.89	2.00 ± 0.71	0.3046
Fibroblasts (H&E)	Border	Placebo laser	1.60 ± 0.55	080 ± 0.45	**0.0161**
		Active laser	1.00 ± 0.00	0.16 ± 0.41	**0.0041**
	Centre	Placebo laser	1.14 ± 0.69	1.14 ± 0.38	> 0.9999
		Active laser	1.60 ± 0.55	0.60 ± 0.55	0.0890
Collagen fibres (MT)	Border	Placebo laser	0.80 ± 0.45	1.00 ± 0.71	0.6213
		Active laser	1.16 ± 0.42	0.33 ± 0.52	**0.0422**
	Centre	Placebo laser	0.71 ± 0.49	0.85 ± 0.38	0.3559
		Active laser	1.00 ± 0.71	0.80 ± 0.84	0.6213

*Note:* Scoring system ranging from 0 to 3. A score of 3 represents the least favourable histological condition, whereas a score of 0 represented the most advanced stage of tissue repair.

*p*‐Values < 0.05 were considered statistically significant (in bold).

^a^ Paired *t*‐test, *n* = 7 patients/group.

**FIGURE 2:**
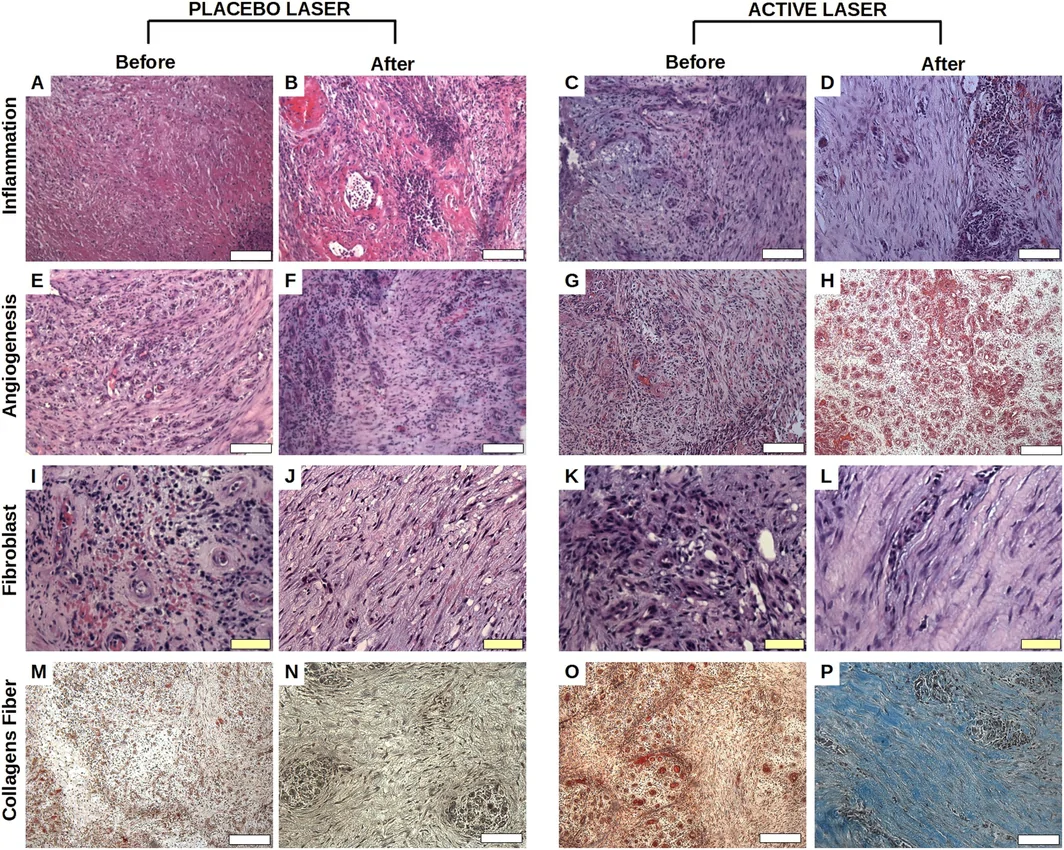
Representative photomicrographs illustrating the histological appearance of the wounds before and after treatment in the placebo laser and active laser groups. Histological scoring was performed directly by microscopic examination of all histological sections under blinded conditions. The images shown are representative fields only and were not used for quantitative or semiquantitative scoring. Images were captured before (A, E, I, M and C, G, K, O) and after 15 treatment sessions (B, F, J, N and D, H, L, P). (A–L) haematoxylin and eosin (H&E) staining. (M–P) Masson's trichrome (TM) staining with collagen fibres in blue. White scale = 100 μm. Yellow scale = 50 μm.

Cellularity scores, reflecting fibroblast density, decreased significantly at the wound edge in both groups (*p* = 0.0161 for AL and *p* = 0.0041 for PL), indicating increased fibroblast proliferation in the periphery (Figure 2C). However, no significant differences were noted in the wound centre. Collagen fibre deposition, assessed using Masson's trichrome staining, showed improved organization at the wound edge in the AL group (*p* = 0.0422) (Figure 2D). Again, no significant changes were noted in the wound centre in either group. Collectively, these findings suggest that laser therapy positively influenced the healing process, particularly, at the wound margins, by modulating inflammation and promoting angiogenesis, fibroblast activity and collagen maturation.

## 4 Discussion

This study aimed to evaluate whether AsGa laser irradiation (904 nm), when combined with conventional wound dressing, can accelerate the healing process of chronic wounds. In a novel approach, the study employed clinical analysis, lesion area measurement, morphological wound assessment and microbiological testing targeting multiple pathogens commonly involved in chronic infections, such as *P. aeruginosa* , *E. coli* , *K. oxytoca* and *P. mirabilis* . Additionally, histopathological analyses were conducted to investigate healing activity at the cellular level. Our findings indicated that laser PBM reduces bacterial colonization and promotes faster lesion retraction. Furthermore, it was effective in stimulating granulation tissue formation and reducing slough, while also promoting fibroblast proliferation, angiogenesis, pain relief and collagen synthesis, all factors that contribute to accelerated wound healing and tissue restoration.

Several experimental studies with AsGa lasers (904 nm) have shown their ability to modulate molecular processes involved in tissue regeneration and support our findings [32, 33, 34]. Santos et al. [35] observed that AsGa laser application promoted muscle regeneration. Moreover, laser therapy accelerated wound contraction, increased collagen deposition, improved extracellular matrix organization and resulted in more efficient tissue regeneration [36, 37, 38, 39]. A key mechanism is the modulation of the inflammatory response, as evidenced by the reduction of inflammatory infiltrates and decreased expression of pro‐inflammatory mediators [40].

Few clinical trials have evaluated the effectiveness of GaAs laser in healing chronic ulcers in humans. Most studies have used other laser types, such as the aluminium gallium indium phosphide (AlGaInP) laser (Po—30 mW, 660 nm), which, over 6 months of treatment, resulted in a greater number of fully healed venous ulcers and fewer recurrences [41]. A recent study by Cardoso et al. with the AsGa laser demonstrated that irradiation at 904 nm, especially at a dose of 10 J/cm^2^, achieved complete healing in 60% of diabetic ulcers after 10 weeks, outperforming lower doses (4 and 8 J/cm^2^) [26]. Miranda et al. also proposed testing various energy densities (4, 8 and 12 J/cm^2^) with a 660 nm HeNe laser to determine optimal parameters for diabetic ulcer treatment [42].

Degerman et al. reported that laser therapy (904 and 635 nm) reduced venous ulcer healing time in elderly patients by an average of 123 days, regardless of prior wound duration [43]. In a systematic review, Santos et al. confirmed that PBM significantly reduced ulcer size (22.96%) and suggested that wavelengths between 632.8 and 685 nm, at doses between 3 and 6 J/cm^2^, were most effective [44]. Together, these studies support the therapeutic potential of PBM, although standardized treatment parameters remain necessary. The present randomized double‐blind trial expands previous clinical evidence by integrating clinical, microbiological and histopathological outcomes, providing complementary information on the biological effects of adjunctive AsGa laser therapy.

The demographic characteristics of our cohort were consistent with previous reports, with a predominance of male participants and a high frequency of venous ulcers associated with systemic hypertension, with or without diabetes mellitus [2, 45, 46, 47, 48, 49].

To evaluate wound contraction, we measured wound edges and calculated total wound area in cm^2^ using ImageJ software. The laser‐treated groups showed greater reductions in wound size compared to the control group. This suggests that phototherapy combined with traditional dressings is effective. Therefore, we also explored other conditions to investigate mechanisms involved in healing, analysing bacterial colonization profiles, inflammatory parameters and collagen deposition.

Experimental studies have shown that this adjunct treatment model can rapidly eliminate bacteria and achieve faster wound healing. We analyzed the bacteria *P. aeruginosa, E. coli, K. oxytoca* and *P. mirabilis* using PCR [50]. These Gram‐negative bacteria were selected because of their frequent association with chronic wound infections [51]. Gram‐negative bacilli possess anti‐phagocytic and adhesion mechanisms and can release endotoxins and exotoxins, which prolong inflammation and hinder bacterial elimination [11]. Guan et al. identified a predominance of Gram‐negative bacteria, especially *P. aeruginosa* and *E. coli*, in wounds lasting over 3 months, which aligns with our findings [52]. In the laser‐treated group, we observed a significant reduction in *P. aeruginosa* , *E. coli* and *P. mirabilis* , while in the control group, only *P. mirabilis* showed a decrease. Carbinatto et al. also reported that laser therapy combined with other treatments accelerated wound healing, reduced bacterial contamination and lowered treatment costs [53]. Experimental evidence suggests that laser irradiation may impair bacterial viability through oxidative stress, membrane disruption and damage to nucleic acids and proteins [54, 55].

The presence of Gram‐negative bacteria is strongly associated with complications in wound healing due to persistent inflammation, delayed collagen deposition and impaired tissue regeneration. Histopathological evaluation demonstrated reduced inflammatory infiltrate, improved angiogenesis scores, greater fibroblast cellularity and more advanced extracellular matrix organization in the ALgroup.

In our study, reduced inflammatory infiltrates and improved collagen deposition were evident in the AL group compared to the placebo group. Initially, both groups had high levels of inflammatory cells. However, over time, the placebo group showed only limited progression to the subsequent healing phases. We also observed more organized connective tissue with dense collagen fibres in the laser group, contrasting with the initial loose, disorganized tissue. In a quantitative and semi‐quantitative analysis, Lima et al. found that PBM‐treated wounds exhibited organized collagen bundles consistent with advanced extracellular matrix remodelling, indicative of the final healing phase [56]. Other studies had similarly demonstrated increased collagen expression in the extracellular matrix following PBM therapy [57]. Despite methodological differences, the superior outcomes associated with PBM are evident in the morphological improvements of scar tissue, supporting our findings.

One limitation of this study is that, although it established a correlation between microbiological and histological data, specific inflammatory mediators and cytokines were not assessed. Analysing these biomarkers could provide a deeper understanding of the cellular and molecular responses involved in modulating inflammation and collagen deposition, allowing for a more detailed characterization of the effects of bacterial infection and therapeutic intervention.

Furthermore, although diabetes mellitus is a well‐recognized modifier of wound healing, the present randomized clinical trial was not statistically powered to evaluate treatment effects according to diabetic status. Therefore, subgroup analyses were not performed, as they would substantially reduce the sample size of each treatment group, decreasing statistical power and increasing the risk of Type II error (false‐negative findings). Future randomized clinical trials specifically designed and adequately powered to investigate treatment responses according to diabetic status are warranted.

Furthermore, some potentially confounding clinical variables were not analyzed in this study, such as parameters associated with diabetes mellitus (glycated haemoglobin levels, perfusion of the affected limb, type of offloading device used, duration of diagnosis and treatment modality), as well as indicators related to venous insufficiency (ankle‐brachial index—ABI). Although this does not compromise the findings observed, including these data could have contributed to a more in‐depth analysis of clinical outcomes and broadened the applicability of the results across different clinical contexts.

Based on the findings of this study, it can be concluded that adjuvant therapy with AsGa laser at 904 nm, when combined with conventional wound dressings, significantly enhances tissue repair and offers clear benefits from both microbiological and histopathological perspectives. This modality has proven to be a safe and effective strategy for managing chronic wounds, accelerating healing while contributing to a reduction in bacterial burden and promoting favourable tissue remodelling. An important strength of this study was the integration of clinical, microbiological and histopathological analyses, providing complementary evidence that adjunctive AsGa laser therapy reduced bacterial burden while promoting tissue repair.

## Author Contributions

**Valéria da Silva Baracho:** conceptualization, formal analysis, investigation, methodology, validation, writing – original draft preparation. **Natália Cristina da Silva:** conceptualization, formal analysis, investigation, methodology. **Lourdes Fernanda Godinho:** formal analysis, investigation, methodology. **Cláudia Aparecida Fernandes Cordeiro:** formal analysis, investigation, methodology. **Lorranne Ferreira Custodio:** investigation. **Brendha Lorrany Alves:** investigation, methodology. **Luiz Otávio Lopes Teixeira:** investigation, methodology. **Rodrigo Pizzini de Souza:** investigation. **Marco Antônio Schetino:** methodology, validation. **Vinícius de Oliveira Ottone:** methodology, validation. **Murilo Xavier de Oliveira:** conceptualization, supervision, validation. **Thabata Coaglio Lucas:** conceptualization, formal analysis, funding acquisition, investigation, methodology, project administration, supervision, validation. **Kinulpe Honorato Sampaio:** conceptualization, formal analysis, funding acquisition, methodology, project administration, supervision, validation, writing – original draft preparation.

## Funding

This study was supported by Fundação de Amparo à Pesquisa do Estado de Minas Gerais (FAPEMIG) (APQ‐00726‐22; APQ‐02018‐24) and Kinulpe Honorato Sampaio is a recipient of Conselho Nacional de Desenvolvimento Científico e Tecnológico (CNPq) (303206/2022‐5). The funding agencies had no role in the study design, data collection, data analysis, interpretation of results, manuscript preparation or decision to publish.

## Conflicts of Interest

The authors declare no conflicts of interest.

## Data Availability

The data that support the findings of this study are openly available in Figshare at https://figshare.com, reference number 10.6084/m9.figshare.29640587.v1.
